# Individualized Treatment of Multidrug-resistant Tuberculosis Using Whole-Genome Sequencing and Expanded Drug-Susceptibility Testing

**DOI:** 10.1093/cid/ciaa526

**Published:** 2020-05-08

**Authors:** Navisha Dookie, Nesri Padayatchi, Richard J Lessells, Cherise L Naicker, Sunitha Chotoo, Kogieleum Naidoo

**Affiliations:** 1 Centre for the AIDS Programme of Research in South Africa (CAPRISA), Nelson R Mandela School of Medicine, College of Health Sciences, University of KwaZulu-Natal Durban, South Africa; 2 South African Medical Research Council-CAPRISA HIV-TB Pathogenesis and Treatment Research Unit, Doris Duke Medical Research Institute, University of KwaZulu-Natal Durban, South Africa; 3 KwaZulu-Natal Research Innovation and Sequencing, College of Health Sciences, University of KwaZulu-Natal, Durban, South Africa; 4 King Dinu-Zulu Hospital Complex, South African National Department of Health, eThekwini Health District, Durban, South Africa

**Keywords:** Mycobacterium tuberculosis, whole genome sequencing, individualised treatment, multi-drug resistant tuberculosis

## Abstract

A case of multidrug-resistant tuberculosis is presented. It highlights the role of whole-genome sequencing, expanded phenotypic drug susceptibility testing, and enhanced case management, offering a more complete understanding of drug susceptibility to *Mycobacterium tuberculosis*. This approach guides an effective individualized treatment strategy that results in rapid sustained culture conversion.

Multidrug-resistant tuberculosis (MDR-TB) treatment has been recently transformed with the introduction of the standardized short-course (SSC) regimens as the preferred option for treatment [1]. MDR-TB is a global health crisis affecting approximately 500 000 individuals annually and is considerably more difficult to treat than drug-susceptible TB disease. Given the magnitude of the disease, the recommended treatment in high-burden settings remains standardized empirical combination regimens. This has been largely driven by the need to scale-up treatment provision coupled with limited access to laboratory-based drug-susceptibility testing (DST) [1, 2]. Rapid molecular-based DST such as Xpert MTB/RIF Ultra (Xpert; Cepheid, USA) and MTBDR*plus* and MTBDR*sl* line probe assays (LPAs; Hain Lifescience GmbH, Nehren, Germany) provides information on a small selection of key drug-resistance mutations. Hence, treatment regimens may contain ineffective and potentially toxic drugs [3]. The recent arrival of the new TB drugs bedaquiline (BDQ) and delamanid holds significant promise in improving drug-resistant TB (DR-TB) treatment success. Clinical trials are currently underway to evaluate the use of these new drugs in various regimens, with the goal of creating shorter, injection-free standardized regimens. These drugs have been incorporated into current regimens since they became available [4]. As a consequence, early reports of resistance to both of these agents are emerging [5]. 

A compelling alternative, presented in the case reported here, is to individualize therapy based on whole-genome predictions of susceptibility. Whole-genome sequencing (WGS) technology for *Mycobacterium tuberculosis* has advanced significantly and has progressed from the research arena to clinical application for diagnosis and management of DR-TB. Leveraging on the advances in WGS technology coupled with enhanced case management by a team of DR-TB clinicians, we portray how the technology can be used to provide personalized care for patients with DR-TB. This approach could potentially impact the prognosis and outcomes of the disease.

## CASE REPORT

**Figure 1.**
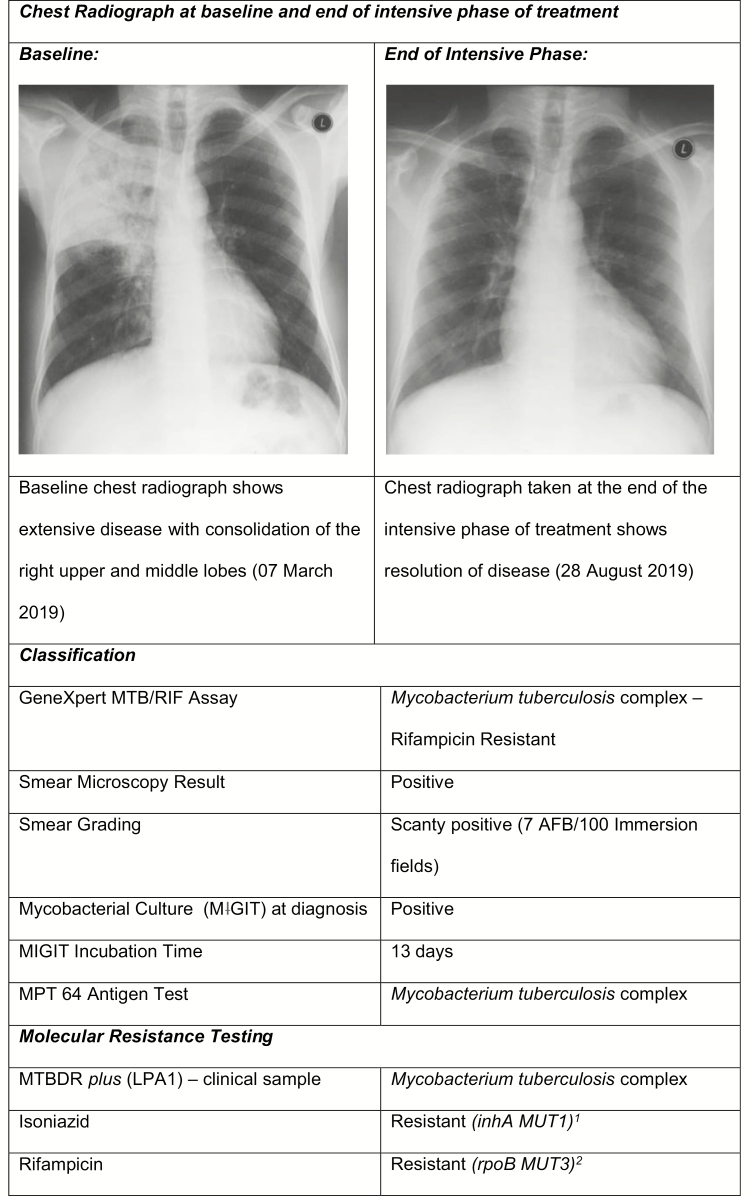

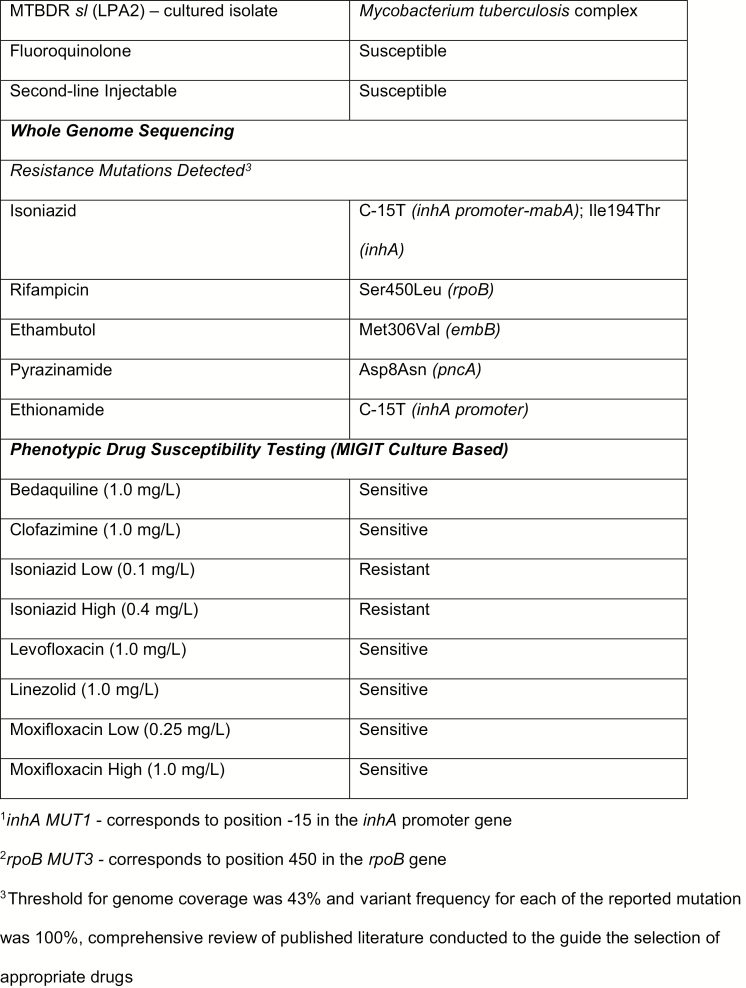
Chest radiographs and complete laboratory profiling of the *Mycobacterium tuberculosis* isolate. Abbreviations: AFB, acid-fast bacilli; Asn, asparagine; Asp, aspartic acid; Ile, isoleucine; Leu, leucine; LPA, line probe assay; Met, methionine; MIGIT, mycobacteria growth indicator tube; MPT, MPT64 protein detection-based immunochomatographic test; MTBDR, MTBDRplus line probe assay; Ser, serine; Thr, threonine; Val, valine.

A 41-year-old male was referred in March 2019 to the specialist DR-TB referral hospital in KwaZulu-Natal, South Africa, with pulmonary rifampicin-resistant TB, diagnosed with the Xpert Ultra assay. The patient was subsequently enrolled into the effectiveness of individualised multi-(extensively) drug-resistant tuberculosis treatment study (CAPRISA 020 InDEX study) and randomized to receive individualized treatment based on WGS of the cultured *M. tuberculosis* isolate [6]. The patient presented with classic clinical features associated with active TB, which included a 2-week history of cough, night sweats, chest pain, weight loss, and poor appetite. Chest radiography indicated consolidation in the right upper lobe and bilateral infiltration of lower zones. His past medical history was notable for a previous episode of drug-susceptible TB in 2004, for which he completed 6 months of treatment. He was diagnosed with human immunodeficiency virus in 2004 and commenced on antiretroviral treatment (ART) since his diagnosis. On presentation, his CD4 T-cell count was 172 cells/μL and viral load was <150 copies/mL. On enrollment into the study, MDR-TB was confirmed by the MTBDR*plus* LPA performed on the sputum sample, demonstrating resistance mutations in *rpo*B and the *inh*A promoter region. No mutations were detected on the MTBDR*sl* LPA, indicating susceptibility to the fluoroquinolones and second-line injectable drugs. He was initiated on a standard, injection-free regimen that contained BDQ (400 mg; loading dose for 2 weeks, 200 mg; 3 doses per week), linezolid (LZD; 600 mg daily), isoniazid (INH) high-dose (INH-HD; 900 mg daily), levofloxacin (LFX; 1 g daily), clofazimine (CFZ; 100 mg daily), pyrazinamide (Z; 1.25 g daily), and ethambutol (E; 1.2 g daily), indicated for 9 months of treatment [7]. His initial ART regimen, which was comprised of tenofovir (300 mg), lamivudine (300 mg), and efavirenz (600 mg), was switched to tenofovir, emtricitabine (200 mg), and nevirapine (200 mg) on commencement of a BDQ-containing regimen, given the interaction between BDQ and efavirenz [8]. LZD was stopped after 1 month due to anemia, corresponding with approximately 25% decrease in hemoglobin from 11.4 g/dL to 8.9 g/dL. His hemoglobin level subsequently increased to 9.7 g/dL. WGS profiling (MiSeq; Illumina V3.0, USA) and bioinformatics analysis (CLC Genomics Workbench v6.0.1, Qiagen, the Netherlands) conducted on the patient’s isolate showed mutations associated with resistance to INH, RIF, Z, E, and ethionamide. As per study protocol, the patient’s WGS results and clinical characteristics were closely reviewed by a panel of local and international drug-resistant TB experts to individualize patient management. Due to the presence of extensive disease, additional INH resistance mutations, and the presence of E and Z resistance, treatment was modified to a regimen of BDQ, LZD (600 mg/daily), LFX, CFZ, and teridizone (750 mg/daily), indicated for 18 months of treatment (6 weeks after initial treatment initiation) [1]. The patient tolerated the reintroduction of LZD (5-week interruption) for the remaining duration of the intensive phase. The patient’s sputum cultures converted to negative at month 2 and remained negative. Figure 1 contains the complete laboratory profiling data (WGS profiling and extended DST) of the patient’s clinical isolate and chest radiographs.

## DISCUSSION

We report on a case of MDR-TB treated using an individualized treatment strategy based on WGS prediction of drug susceptibility to the infecting organism. The case highlights the challenges associated with the lack of appropriate diagnostics to guide the use of the standard MDR-TB regimen. The application of WGS, expanded phenotypic DST, and enhanced case management revealed that the patient was on a regimen that contained 3 drugs with confirmed susceptibility. This included BDQ, LFX, and CFZ, as LZD was discontinued after 1 month of treatment due to anemia. Further, the patient would have received only 2 effective drugs (LFX and CFZ) during the continuation phase of treatment. WGS profiling demonstrated conventional resistance-associated mutations in the *inhA* promoter region and *rpoB* gene regions concordant with LPA results. In the case of INH, an additional mutation was detected in the *inhA* coding region, which is not available on LPA. A combination of the mutations in the promoter and coding regions of the *inhA* gene results in highly variable minimum inhibitory concentrations (MICs), ranging from 0.5 to 2.0 mg/L [9, 10]. LPA fails to predict the precise level of resistance in cases where additional mutations not present on the test raises the MIC of the isolate. Phenotypic testing confirmed high-level INH resistance. The role of INH-HD in the treatment of MDR-TB and in patients with different genetic variants of INH-resistant TB has yet to be determined. In the absence of this evidence, quantifying the level of INH resistance by phenotypic DST is advised. Given the high background burden of resistance to E and Z, with approximately 50% of MDR-TB patients with documented resistance to Z and 61% to E [11], replacement of these agents with similar sterilizing agents is warranted. This highlights that careful selection of effective companion drugs is warranted as these potentiate the activity of core drugs promoting relapse-free cure. Further, the use of an SSC regimen should be closely monitored, especially when 1 or more of group A drugs are clinically contraindicated, potentially compromising the regimen. In this case, modification of the drug regimen was required because the patient developed anemia.

DR-TB treatment is rapidly transitioning into shorter, injection-free standard regimens that include the novel 6-month combination of BDQ, LZD, and pretomanid for extensively DR-TB and complicated cases of MDR-TB, as well as various novel combinations currently under evaluation [12]. The current case highlights that the eligibility criteria for these regimens require careful consideration and should ideally be guided by individual-level DST and clinical profiling. In this report, WGS and expanded DST were used as diagnostic adjuncts, demonstrating inadequacy of the novel injection-free BDQ-containing regimen. A significant number of DR-TB patients now receive BDQ-containing treatment regimens; however, as a result of increased use of the drug, there are emerging reports of acquired BDQ resistance with cross-resistance to CFZ [5]. This has important implications for the implementation of novel regimen combinations. At present, no rapid assay has the capability of detecting resistance to BDQ, LZD, CFZ, and cycloserine, which are key components of DR-TB [13]. While robust sequencing technology is available, elucidation of the genetic basis for resistance to the newer drugs remains limited and thus warrants confirmation by phenotypic DST in the interim, especially in cases with prior exposure to these drugs.

In conclusion, the case highlights the shortfalls of current diagnostic platforms in guiding DR-TB treatment. Initiation of treatment based on the standard diagnostic pipeline may inadvertently result in patients receiving suboptimal treatment, amplify resistance, and increase the risk of DR-TB transmission. Large-scale studies to assess the role of diagnostic adjuncts such as WGS, targeted sequencing panels, and expanded phenotypic assays are urgently needed in order to determine adequate treatment selection and personalized care approaches as highlighted in the described case. We strongly support the recommendations by Dowdy et al to address the policy gap regarding implementation of standard regimens. DST should be conducted with at least the core drugs such as BDQ and LZD, and no patient should be continued on the regimen for more than 2 months without documented susceptibility to these agents. Further, prior to the continuation phase, patients should have documented susceptibility to the fluoroquinolone and other key drugs carried into the continuation phase [14]. While standardized treatment approaches improve treatment access at a reduced cost and complexity, we cannot ignore the potential risk of resistance amplification.

## Notes

***Acknowledgments.*** The authors thank the laboratory staff for their assistance with handling, processing, and analyzing specimens at the National Institute of Communicable Diseases–Centre for Tuberculosis and the CAPRISA 020 InDEX study team.

***Financial support.*** This work was supported by the European and Developing Countries Clinical Trials Partnership Fellowship (TMA2018CDF-2372 and TMA2018SF-2476).

***Potential conflicts of interest.*** The authors: No reported conflicts of interest. All authors have submitted the ICMJE Form for Disclosure of Potential Conflicts of Interest.
